# Circular RNAs in Diabetic Nephropathy: Updates and Perspectives

**DOI:** 10.14336/AD.2022.0203

**Published:** 2022-10-01

**Authors:** Miao Liu, Junli Zhao

**Affiliations:** Department of Nephrology, Shanghai University of Medicine & Health Sciences Affiliated Zhoupu Hospital, Shanghai 201318, China.

**Keywords:** circular RNA, diabetic nephropathy, miRNA sponge, fibrosis

## Abstract

Circular RNAs (circRNAs) are widespread endogenous transcripts lacking 5′-caps and 3′-polyadenylation tails. Their closed-loop structure confers exonuclease resistance and extreme stability. CircRNAs play essential roles in various diseases, including diabetes. Diabetic nephropathy (DN) is the leading cause of end-stage kidney disease and is one of the most common complications of diabetes. CircRNAs are key in DN and therefore important for understanding DN pathophysiology and developing new therapeutic strategies. In the present review, we briefly introduce the characteristics and functions of circRNAs and summarize recent discoveries on how circRNAs participate in DN. Based on these advances, we suggest future perspectives for studying circRNAs in DN to improve DN treatment and management.

The prevalence of diabetes has recently increased significantly, and 11.6% of the Chinese adult population is estimated to have diabetes [1]. The high overall prevalence of diabetes, and the increased risks of microvascular and macrovascular complications, represent an economic and healthcare burden globally [2]. The structural and functional performance of the kidney degenerates with aging, which can be accelerated in patients with diabetes mellitus [3]. Diabetic nephropathy (DN) is one of the most common non-communicable chronic disease complications of diabetes and is characterized by albuminuria and the progressive loss of kidney function [4].

Circular RNAs (circRNAs) are covalently closed RNA loops that originate from pre-mRNAs via back-splicing. Unlike linear RNAs, circRNAs are characterized by the absence of free 5ʹ-caps or 3ʹ-tails. CircRNAs were initially considered byproducts of genetic transcription and splicing and were thought to have no biological function. However, recently, circRNAs have been shown to play vital roles in multiple diseases, especially in diabetes mellitus [5-10]. The circRNA CDR1as targets Myrip to regulate insulin granule secretion; it also targets Pax6 to enhance insulin transcription via the CDR1as/miR-7 pathway [11]. Stoll et al. identified the circRNA generated from an intron of the insulin gene, namely, ci-Ins2/ci-INS, that interacts with the RNA-binding protein (RBP) TAR DNA-binding protein 43-kDa (TDP-43) at the transcriptional level to regulate insulin secretion [12]. In addition, ci-Ins2/ci-INS expression levels are downregulated in the islets of rodents and humans with type 2 diabetes (T2D) [12]. Furthermore, circPPM1F can modulate M1 macrophage activation and pancreatic islet inflammation in type 1 diabetes mellitus through the circPPM1F-HuR-PPM1F-NF-κB axis [6]. In human umbilical vein endothelial cells (HUVECs) cultured under high glucose (HG) conditions, hsa_circ_0054633 protects against HG-induced endothelial cell dysfunction by targeting the miR-218-ROBO1/HO-1 axis [13].

Approximately, 40% of the patients with diabetes suffer from DN—a leading cause of death in such patients [14, 15]. Investigations into the dysregulated expression profiles and roles of circRNAs in DN will improve our understanding of DN pathogenesis and may provide new therapeutic strategies. Currently, there is an increasing number of reports on the functions and mechanisms of dysregulated circRNAs in DN (Fig. 1). In the present review, we briefly summarize the features of circRNAs and discuss the current understanding of their roles in DN and the underlying mechanisms of action. We provide future perspectives for research, which is expected to uncover novel therapeutic targets that could eventually lead to developing effective DN treatment or management strategies.

**Figure 1.**
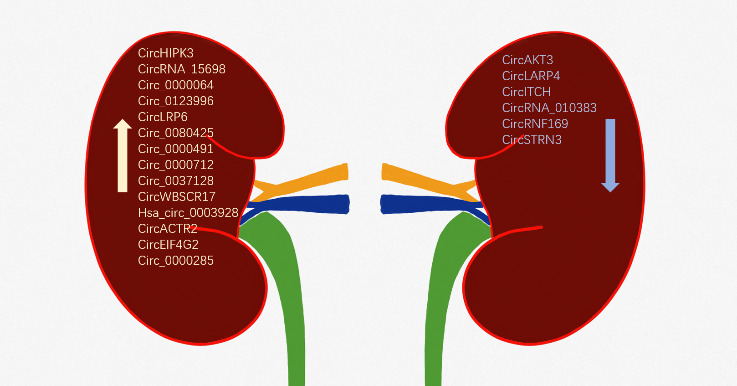
The upregulated and downregulated circRNAs in diabetic nephropathy.

## Characteristics of circRNAs

CircRNAs are a relatively newly described type of RNA characterized by several features. Their distinguishing circular structure lacks free 5′ and 3′ ends; therefore, they are remarkably tolerant to exonuclease degradation. Owing to their stability, the half-life of circRNAs is prolonged compared to that of linear RNAs [16]. For example, in plasma, the average half-life of circRNAs is more than 48 h, whereas that of linear mRNAs is approximately 10 h [17, 18]. Thus, circRNAs can be precisely detected in body fluids including saliva, plasma, and urine [19-21].

Stability also leads to accumulation; therefore, circRNAs are remarkably abundant. The level of circRNAs may be more than 10-fold that of their corresponding linear transcripts [22, 23]. This phenomenon is more prominent in non-proliferating cells of the neural tissue, especially in the brain [22, 24, 25].

CircRNAs are not only widely expressed in different organisms, including plants [26], mice [27], and humans [28], but they are also highly conserved between species [22, 29]. Among the circRNAs generated in human fibroblasts and murine testis, 69 are circularized at exactly homologous start and stop points; their encoding genes constitute approximately 15% of the genes producing circRNAs in both species [23]. Approximately, 20% of the splice sites involved in porcine circRNA biogenesis are also functionally conserved between mice and humans [30]. The regulatory role of RBPs on circRNA biogenesis is also conserved; the conserved modulation of the biogenesis of circRNAs by FUS in mice and humans is one such example [31, 32].

CircRNAs generally exhibit cell-, tissue-, developmental stage-, and disease-specific expression patterns [30, 33-35]. For example, circSRY is highly transcribed in the mouse testis but not in other tissues [36]. In humans, circRNAs are more abundant in the brain than in other tissues [25] and in fetal tissues than in the corresponding adult tissues [37]. The cellular localization of circRNAs is also characteristic. Based on their structural sequences, circRNAs can be classified into three types: exonic circRNAs (ecircRNAs), consisting of exons, mainly located in the cytoplasm, and functioning as competing endogenous RNAs (ceRNAs); circular intronic RNAs (ciRNAs), consisting of introns; and exon-intron circRNAs (EIciRNAs), consisting of both exons and introns. Both ciRNAs and EIciRNAs are mainly located in the cell nucleus [38]. Importantly, the expression patterns of circRNAs are disease-specific [39, 40]. This feature, together with their stability and abundance, has drawn the attention of researchers aiming to identify circRNAs as biomarkers with clinical utility [17, 41, 42]. For example, a novel circRNA, F-circEA, generated from the echinoderm microtubule-associated protein-like 4 (*EML4*)-anaplastic lymphoma kinase (*ALK*) fusion gene plays an oncogenic role in non-small cell lung cancer (NSCLC) by activating ALK kinase [43]. F-circEA in the plasma of patients with the *EML4-ALK* translocation has been proposed as a novel liquid biopsy biomarker for NSCLC [43].

## Functions of circRNAs

The various biological functions of circRNAs include behaving as miRNA or lncRNA sponges [44, 45], acting as protein decoys or scaffolds for RBPs [32, 46], regulating transcription and splicing processes [47, 48], and serving as translation templates [49, 50].

The circRNAs that located in the cytoplasm mainly function as miRNA sponges, sequestering target miRNAs from downstream mRNAs and thus regulating gene expression at the post-transcriptional level [51]. The most well-known circRNA is CDR1as, which harbors more than 70 conserved seed targets of miR-7 [52]. By binding to miR-7, CDR1as modulates the expression of miR-7 target mRNAs that participate in diverse diseases, including diabetes mellitus and various cancers [52]. Another well-known circRNA is circSRY, containing 16 binding sites for miR-138, that functions as a miR-138 sponge in the mouse testis [36, 44]. As individual circRNAs usually harbor binding sites for many miRNAs, they can have diverse functions under different conditions, which is consistent with their cell- and tissue-specific expression. For example, circMTO1 can bind miR-6893, miR-630, miR-221, and miR-9 in different cancers, where it can play oncogenic or tumor-suppressor roles depending on the cellular context [53].

CircRNAs can also bind and sequester proteins, serving as decoys and scaffolds for RBPs [54]. For example, circAmotl1 binds to STAT3 and functions as a decoy, facilitating its nuclear translocation. Nuclear STAT3 upregulates DNMT3a expression and promote wound healing [55]. Du et al. found that circFoxo3 binds to p21 and CDK2 to block the cell cycle by functioning as a scaffold and forming a ternary complex [56]. CircFoxo3 can also bind to anti-senescence and anti-stress proteins to participate in cellular senescence [57]. CircACC1 can stabilize and stimulate the enzymatic activity of the AMPK holoenzyme by binding to its regulatory β and γ subunits and forming a ternary complex, thus participating in metabolic reprogramming under metabolic stress [58]. Similarly, circ-CUX1 binds to EWS RNA-binding protein 1 (EWSR1) to promote its interaction with MYC-associated zinc finger protein (MAZ), transactivating MAZ, and modulating CUX1 expression to facilitate the metabolic reprogramming and progression of neuroblastoma (NB) [59].

CircRNAs are also able to modulate gene expression at the transcriptional and post-transcriptional levels. CircRNAs can mediate the expression of genes by regulating their transcription and splicing processes. Nuclear ciRNAs and EIciRNAs *cis*-regulate the transcription of their parental genes [60, 61]. Ci-ankrd52 and ci-sirt7 accumulate at transcription sites and interact with polymerase II to positively regulate the transcription of their parental genes [61]. In contrast, some circRNAs can compete with the mRNA splicing of their parental genes by occupying splice sites [48, 62]. For example, MBL can induce the circularization of its parental gene to produce circMBL at the cost of the generation of linear MBL [48]. If the canonical splicing efficiency for generating mRNA is increased, circRNA biogenesis by back-splicing slows down [48]. At the post-transcriptional level, circRNAs can control gene expression by sponging specific miRNAs. For example, circZNF532 sponges miR-29a-3p and increases the expression of NG2, LOXL2, and CDK2, thus regulating diabetes-induced retinal pericyte degeneration and vascular dysfunction [63].

Finally, several circRNAs are translated in cap-independent manners, including internal ribosome entry site (IRES) element-dependent and N^6^-methyladenosine (m^6^A) modification-dependent pathways [64]. For example, circβ-catenin has an IRES sequence; it is translated into a novel 370-amino acid β-catenin isoform that activates the Wnt pathway and promotes liver cancer cell growth [65]. The m^6^A residues in circRNAs are recognized by YTHDF3, inducing the cap-independent translation of such circRNAs, in collaboration with the translation initiation factors eIF4G2 and eIF3A [66]. Approximately, 13% of all circRNAs are estimated to contain m^6^A modifications, and even a single m^6^A site can enable initiation of artificial circRNA translation [66].

## Roles of circRNAs in DN

### CircRNAs in mesangial cells

#### CircHIPK3

CircHIPK3 (hsa_circ_0000284) is a well-known circRNA that is driven by the host gene *HIPK3* and is involved in various diseases, including cancers [67, 68] and diabetes [69]. Shan et al. recently showed that circHIPK3 expression is significantly increased in diabetic retinas. The expression of circHIPK3 in human retinal vascular endothelial cells was upregulated in a time-dependent manner after HG treatment *in vitro*, and circHIPK3 is capable of upregulating the expression of vascular endothelial growth factor-C, FZD4, and WNT2 by sponging miR-30a-3p, thus resulting in endothelial over-proliferation and vascular dysfunction [69]. In mesangial cell lines, the expression of circHIPK3 increases in a time-dependent manner under HG conditions. Furthermore, *in vivo* studies have suggested that circHIPK3 expression is upregulated during DN progression. Silencing circHIPK3 suppresses the proliferation of mesangial cell lines and the expression of TGF-β1, collagen type I (Col-I), and fibronectin (FN); this phenotype can be rescued by miR-185 inhibition. A luciferase assay results indicated that miR-185 directly targets wild-type circHIPK3. Thus, the upregulation of circHIPK3 expression resulting from HG exposure might promote the progression of DN by regulating miR-185, leading to the proliferation of mesangial cells and the accumulation of extracellular matrix (ECM) [70]. However, when HUVECs were cultured under HG conditions, the expression of circHIPK3 decreased in a dose- and time-dependent manner. In addition, circHIPK3 levels were significantly decreased in human aortic endothelial cells (HAECs) from diabetic patients compared to those in healthy controls [71]. Ectopic circHIPK3 overexpression or miR-124 inhibition could significantly inhibit HG-induced viability reduction and the promotion of apoptosis in HUVECs, suggesting that there is a potential protective role of circHIPK3/miR-124/SphK1 and the STAT3 axis against diabetic-associated vascular injury [71].

#### CircRNA_15698

Microarray analysis and quantitative reverse transcription-polymerase chain reaction (qRT-PCR) verified that the expression of circRNA_15698 (mmu_circ_0015698) was significantly upregulated in *db/db* DN mice compared with that in *db/m* non-DN mice, and *in vitro* studies in mouse mesangial cells showed that circRNA_15698 levels increased under HG conditions in a time-dependent manner [72]. The expression levels of fibrosis-related proteins, including FN, Col-I, and collagen type IV (Col-IV), were higher in *db/db* DN mice and mouse mesangial cells under HG conditions than in *db/m* non-DN mice and mouse mesangial cells under normal glucose (NG) conditions, respectively. Silencing circRNA_15698 significantly decreased the expression of FN, Col-I, and Col-IV, which could be rescued by miR-185 inhibition. The cytoplasmic location of circRNA_15698 suggests that it might function by sponging miRNA. This was further validated by demonstrating the molecular binding between miR-185 and circRNA_15698. TGF-β1 expression is negatively correlated with miR-185 expression in *db/db* DN mice, and the upregulation of TGF-β1 expression induced by inhibiting miR-185 could be reversed by silencing circRNA_15698. A luciferase reporter assay was used to demonstrate the direct interactions between TGF-β1 and miR-185. In conclusion, circRNA_15698 might promote the biosynthesis of ECM-related proteins through the circRNA_15698/miR-185/TGF-β1 axis [72].

#### CircAKT3

The expression of circAKT3 was significantly decreased in *db*/*db* mice than in matched normal *db*/*m* mice, and *in vitro* studies in mouse mesangial cells showed that circAKT3 levels decreased remarkably under HG conditions in a time-dependent manner [73]. Under HG stimulation, upregulated FN, Col-I, and Col-IV expression levels were significantly decreased after overexpression of circAKT3. These results suggest that circAKT3 could inhibit ECM accumulation in mouse mesangial cells. In addition, the upregulation of circAKT3 expression inhibited the apoptosis of mesangial cells. MiR-296-3p expression was significantly higher in *db/db* DN mice than in normal *db/m* mice and was negatively correlated with circAKT3 expression in *db/db* DN mice. However, E-cadherin expression was significantly lower in *db/db* DN mice than in normal *db/m* mice and was positively correlated with circAKT3 expression in *db/db* DN mice. A luciferase reporter assay was used to validate the direct interactions of miR-296-3p with E-cadherin and circAKT3. Overexpression of miR-296-3p abolished the suppressive effects of circAKT3 on the apoptosis of mouse mesangial cells and the expression of ECM-related proteins, including FN, Col-I, and Col-IV, which could be rescued by co-transfection with E-cadherin [73]. These results suggest that circAKT3 plays a protective role in DN by inhibiting mouse mesangial cell apoptosis and suppressing ECM accumulation through the regulation of the miR-296-3p/E-cadherin signaling pathway. CircAKT3 is therefore a potential therapeutic target for DN.

#### Circ_0000064

Circ_0000064 (hsa_circ_0000064) is oncogenic in hepatocellular carcinoma and lung cancer [74, 75]. Ge et al. found that the expression of circ_0000064 increased significantly after HG stimulation in mouse mesangial cells [76]. Knockdown of circ_0000064 increased miR-143 expression, while inhibiting the expression of Col-I, Col-IV, and FN, which could be rescued by miR-143 inhibitors. Similarly, the suppression of cell proliferation and promotion of apoptosis induced by circ_0000064 knockdown could be reversed by inhibiting miR-143. Therefore, circ_0000064 participates in kidney fibrosis in patients with DN by aggravating the fibrosis of mesangial cells via sponging miR-143, thus promoting the expression of fibrosis-associated proteins, including Col-I, Col-IV, and FN [76].

#### Circ_0123996

The expression of circ_0123996 increased in a time-dependent manner in mouse mesangial cells treated with HG and increased significantly in patients with T2D and DN compared with that in patients with T2D and no DN and in mice with DN compared with that in normal mice. Circ_0123996 is therefore a candidate biomarker for DN. Knockdown of circ_0123996 inhibited the proliferation of mesangial cells, which could be partly rescued by miR-149-5p inhibition. Luciferase reporter assays and pull-down assays were used to validate the direct interactions of miR-149-5p with circ_0123996 and Bach1. The expression of fibrosis-related proteins, including FN and Col-IV, could be inhibited by suppressing circ_0123996 [77]. Further mechanistic research has suggested that circ_0123996 enhances the proliferation of mesangial cells and the production of ECM-related proteins by modulating the miR-149-5p/Bach1 axis, thereby promoting the progression of DN [77].

#### CircLRP6

High mobility group box-1 (HMGB1) is a ubiquitous nuclear protein that maintains DNA structure in various cell types and can be released in response to hyperglycemia in diabetes [78]. HMGB1 participates in the development and progression of DN by promoting inflammation [78]. By binding to its receptors toll-like receptor 2 (TLR2) and TLR4, and to advanced glycation end products (RAGE), HMGB1 can initiate cellular signals, such as the NF-κB signaling pathway, and trigger a proinflammatory response [79, 80]. The expression of TLR4 and HMGB1 is upregulated in the kidneys of patients with DN [81]. The expression and functions of HMGB1, and the ceRNA network modulating it in DN, were investigated [82]. Compared with under NG conditions, HG stimulation significantly enhanced the expression of HMGB1, TLR4, and p-NF-κB p65 in mouse mesangial SV40-Mes13 cells. Chen et al. demonstrated that HMGB1 could bind to TLR4 to enhance the phosphorylation, nuclear translocation, and DNA-binding activities of NF-κB in mesangial cells, which could be abolished by glycyrrhizin (an HMGB1 inhibitor) or CLI-095 (a TLR4 inhibitor) [82]. Mechanistic studies have shown that the inhibition of HMGB1 attenuated HG-induced proliferation, oxidative stress, secretion of ECM proteins (FN, Col-IV), and expression of inflammatory cytokines (IL-6, IL-1β, and TNF-α) in mesangial cells via the TLR4/NF-κB pathway. These changes could also be achieved by miR-205 overexpression and abrogated by the upregulation of HMGB1 expression.

The results of a dual luciferase assay suggested that both HMGB1 and circLRP6 could directly target miR-205. Both miR-205 and circLRP6 regulate the phosphorylation of NF-κB p65. CircLRP6 knockdown might protect mesangial cells against HG induction injury, which could be reversed by miR-205 inhibition. Thus, circLRP6 might participate in the pathogenesis of DN via the miR-205/HMGB1 axis and downstream TLR4/NF-κB pathway, which play important roles in the dysregulation of mesangial cells [82]. Considering the effectiveness of blocking HMGB1 for attenuating DN, its upstream regulator circLRP6 could serve as a more efficient therapeutic target for DN [79].

#### CircLARP4

Derived from exons 9 and 10 and the intermediate long intron of the La-related protein 4 (LARP4) gene, circLARP4 expression was significantly downregulated in mesangial SV40-Mes13 cells cultured under HG conditions compared to under control conditions [83]. CircLARP4 overexpression decreased the proliferative ability of mesangial cells, enhanced cell apoptosis, and decreased the expression levels of fibrosis-related markers, including FN, Col-I, and Col-IV. In addition, miR-424 expression levels were downregulated with overexpression of circLARP4. After co-transfection with miR-424 mimics, the ability of cultured mesangial cells to overexpress circLARP4 was partly ameliorated [83]. Thus, circLARP4 may participate in DN development by regulating miR-424 *in vitro*. However, to better understand the roles of circLARP4 in DN, more *in vivo* studies should be performed.

#### Circ_0080425

Circ_0080425 expression was significantly upregulated in a mouse DN model and was positively correlated with the severity of DN [84]. In addition, the expression level of circ_0080425 in cultured mesangial cells increased under HG conditions in a time- and concentration-dependent manner. In contrast, the expression level of miR-24-3p was negatively correlated with the severity of DN. Knockdown of circ_0080425 inhibited the proliferation of mouse mesangial cells and arrested the cells in the S and G0/1 phases. The expression levels of FN, Col-IV, and TGF-β1 were downregulated in knockdown circ_0080425 mesangial cells, which also inhibited ECM generation and accumulation, indicating that circ_0080425 plays essential roles in renal cell fibrosis. Considering the ceRNA function of circRNAs, the direct interactions between circ_0080425 and miR-24-3p were validated using a biotin-coupled miR-24-3p probe and a circ_0080425-specific probe followed by qRT-PCR, as well as by luciferase reporter assays. The negative effects of circ_0080425 knockdown on cell proliferation and fibrosis could be rescued by miR-24-3p inhibitors. A luciferase reporter assay suggested that miR-24-3p could target FGF11, and the effects of the miR-24-3p inhibitor on cell proliferation and fibrosis-related proteins could be almost completely rescued by si-FGF11. Thus, circ_0080425 might promote proliferation and cell cycle progression and upregulate the expression of FN, Col-IV, and TGF-β in mesangial cells by regulating the miR-24-3p/FGF11 axis in DN [84]. The positive correlation between circ_0080425 expression and DN severity highlights its potential as a DN prognostic biomarker, which should be validated in larger cohorts in the future.

#### Circ_0000491

A total of 40 circRNAs were found to be differentially expressed in the DN mouse kidney cortex compared to normal controls, including 18 upregulated and 22 downregulated circRNAs [85]. Among the dysregulated circRNAs, circ_0000491 (mmu_circrna_0000491, chr13: 94111710-94126034), which is generated from the Homer1 gene locus, was upregulated in the DN mouse kidney cortex, and its expression levels in mesangial SV40-Mes13 cells increased following HG exposure in a time-dependent manner [85]. The expression of epithelial-to-mesenchymal transition (EMT) and fibrosis-associated proteins, including vimentin, FN, α-SMA, Col-I, III, and IV, decreased, while the levels of E-cadherin were markedly increased following knockdown of circ_0000491 in HG-treated mesangial cells. However, the levels of EMT and fibrosis-associated proteins were upregulated while those of E-cadherin were downregulated in HG-cultured cells, which is consistent with the expression patterns observed in kidney tissues from mice with DN. The expression of circ_0000491 and miR-101b could be regulated by each other [85]. In addition, both circ_0000491 and TGFβRI could be targeted by miR-101b directly. A further rescue study has suggested that circ_0000491 could promote EMT, ECM accumulation, and fibrosis in mesangial cells by regulating the miR-101b/TGFβRI axis [85].

#### Circ_0000712

The expression of circ_0000712 (mmu_circrna_0000712) has been shown to be upregulated in *db/db* DN mice compared with that in normal *db*/*m* mice [85, 86]. In HG-cultured mesangial cells, the expression levels of circ_0000712 increase in a time-dependent manner. Besides, circ_0000712 silencing suppresses HG-induced apoptosis, inflammation, oxidative stress, and fibrosis in mesangial cells, resulting in the downregulation of IL-1β, IL-6, TNF-α, and the fibrosis-related proteins FN, Col-I, and Col-IV expression, decreased ROS generation and LDH activity, and increased SOD activity *in vitro*. The introduction of miR-879-5p inhibitors could counteract these changes. The predicted binding relationship between miR-879-5p and circ_0000712 or SOX6 was verified by luciferase reporter assays and RNA immunoprecipitation (RIP) assays. The inhibitory effects of miR-879-5p on apoptosis, inflammation, oxidative stress, and fibrosis in mesangial cells could be rescued by co-transfection with SOX6. Circ_0000712 could therefore modulate HG-induced mesangial cell injury, including apoptosis, inflammation, oxidative stress, and fibrosis, by regulating the miR-879-5p/SOX6 axis [86].

#### Circ_0037128

The expression levels of circ_0037128 were significantly elevated in a mouse DN model and in human patients with DN compared with those in the corresponding controls [87]. Circ_0037128 expression was upregulated in HG-induced human mesangial cells in a time- and concentration-dependent manner. Knockdown of circ_0037128 suppressed the proliferation of human mesangial cells and arrested them in the S phase [87]. Furthermore, the synthesis of FN and TGF-β at the mRNA and protein levels was significantly inhibited by circ_0037128 silencing, which could be antagonized by miR-17-3p inhibition. Pull down assays validated that miR-17-3p could be captured by a circ_0037128 probe and that circ_0037128 could be enriched by a miR-17-3p probe, and their direct interaction was further validated using a luciferase reporter assay. Moreover, a co-transfection experiment demonstrated that AKT3 inhibition could reverse the effects of a miR-17-3p inhibitor on cell proliferation and the expression of fibrosis-related proteins, and a luciferase reporter assay further validated their direct interaction [87]. The expression of AKT3 can be regulated by both circ_0037128 and miR-17-3p, which counteract each other at the transcriptional and translational levels. Overall, circ_0037128 provokes DN progression by regulating the miR-17-3p/AKT3 axis to influence mesangial cell proliferation and the accumulation of fibrosis-related proteins. The *in vivo* functions of the circ_0037128/miR-17-3p/AKT3 axis should be further studied.

#### CircITCH

Recently, Zhou et al. validated the downregulation of circITCH expression in retinal pigment epithelial cells isolated from diabetic rats compared to that in normal rats and confirmed that circITCH regulates the process of diabetic retinopathy by modulating miR-22 [88]. To explore the role of circITCH in DN, its expression levels and functions were investigated. Similar to the results in retinal cells, the expression levels of circITCH were downregulated in HG-induced rat mesangial cells [89]. *In vitro* studies showed that when circITCH was overexpressed, the viability and migration of mesangial cells and the expression levels of fibrosis-related proteins (Col-I, α-SMA, and FN) and inflammatory factors (IL-6, IL-1β, and TNF-α), were significantly suppressed [89]. These reductions could be reversed *in vitro* by overexpressing miR-33a-5p or by SIRT6 knockdown. Besides, circITCH was present at higher concentrations in the cytoplasm than in the nucleus, implying that it might serve as a miRNA sponge. Luciferase reporter assays were used to validate the direct interactions between miR-33a-5p and circITCH or SIRT6 [89]. In a streptozotocin (STZ)-induced diabetic mouse model, circITCH overexpression decreased blood glucose level and increased insulin expression. In addition, the upregulated levels of the serum renal function indicators BUN and Scr that were induced by STZ were neutralized by overexpressing circITCH *in vivo* [89]. Furthermore, the pathological injury, fibrosis, and high levels of inflammatory factors in renal tissues observed in a diabetic mouse model were alleviated by intravenously transfecting mouse with circITCH through the tail vein [89]. Thus, both *in vitro* and *in vivo* studies support the targeting of circITCH, which partly functions by regulating the miR-33a-5p/SIRT6 axis, as a promising treatment strategy for DN. Considering the beneficial therapeutic effects of circITCH in mouse models, more *in vivo* studies and clinical trials are warranted to explore the effects of circITCH in patients with DN.

#### CircRNA_010383

Among 140 differentially expressed circRNAs (with fold-changes >2.0) between the kidneys of *db*/*db* mice and the kidneys of their littermates, circRNA_010383 expression was downregulated in diabetic mice compared with that in control mice [15]. The downregulation of circRNA_010383 expression was also confirmed in kidney tissues from patients with DN compared with that in normal kidney tissues. *In vitro* experiments suggested that HG downregulated circRNA_010383 expression in mouse glomerular mesangial cells and mouse tubular epithelial cells. RNA fluorescence *in situ* hybridization indicated that circRNA_010383 and miR-135a are predominantly colocalized in the cytoplasm. The direct interaction between circRNA_010383 and miR-135a was further validated using luciferase reporter assays and pull-down assays [15]. Overexpression of circRNA_010383 in mesangial cells and tubular epithelial cells suppressed the synthesis of Col-I, FN, and α-SMA that was induced by HG and restored the expression of TRPC1 that was suppressed under such conditions, which could be abrogated by co-transfection with miR-135a mimics or TRPC1 siRNA. These results suggest that circRNA_010383 inhibits ECM accumulation in mouse glomerular mesangial and tubular epithelial cells, preventing the progression of glomerular sclerosis and tubulointerstitial fibrosis via the miR-135a/TRPC1 axis.

When circRNA_010383 was overexpressed *in vivo* in *db*/*db* mouse kidneys with an ultrasound-microbubble-mediated gene transfer technique, TRPC1 expression was restored, and ECM protein synthesis was reduced. At the same time, the accumulation of mesangial matrix and the thickness of the glomerular basement membrane decreased, and microalbuminuria severity were alleviated with circRNA_010383 overexpression *in vivo* [15]. These *in vitro* and *in vivo* results suggest that circRNA_010383 is an effective therapeutic target to inhibit proteinuria and renal fibrosis in DN.

### CircRNAs in tubular cells

#### CircWBSCR17

In contrast to the numerous studies focusing on the role of circRNAs in mesangial cells, there are relatively few studies on kidney tubular cells in DN progression. The expression levels of circWBSCR17 in kidney cortex tissue from DN mice and HG-cultured HK-2 cells were remarkably elevated compared with those in healthy mice and normally cultured HK-2 cells, respectively [90]. The suppressed proliferative ability of HK-2 cells caused by the HG treatment could be reversed by knocking down circWBSCR17 and further inhibited by the overexpression of circWBSCR17 *in vitro*. HG-induced HK-2 cell apoptosis, inflammatory cytokine (TNF-α, IL-6, and IL-1β) secretion, and fibrosis-related protein (FN, Col-IV, and Col-I) synthesis were suppressed by silencing circWBSCR17 but were promoted by circWBSCR17 overexpression *in vitro* [90]. Rescue experiments revealed that the effects of knockdown and overexpression of circWBSCR17 could be reversed by inhibiting and overexpressing miR-185-5p, respectively. RIP and luciferase reporter assays have demonstrated the target association between circWBSCR17 and miR-185-5p. MiR-185-5p expression levels were negatively correlated with circWBSCR17 expression and SOX6 expression in DN mice [90]. The function of miR-185-5p could be rescued by SOX6 *in vitro*, and their direct interaction was validated using a luciferase reporter assay. Thus, circWBSCR17 controls the expression of SOX6 by absorbing miR-185-5p to aggravate the apoptosis, inflammatory response, and fibrosis of human kidney tubular cells under HG treatment *in vitro* [90]. Further studies focusing on the physiological functions of circWBSCR17 *in vivo* are needed.

#### Hsa_circ_0003928

A study focusing on tubular cells was conducted recently by An et al. [91]. They determined that the expression levels of hsa_circ_0003928 were significantly increased in the serum of patients with DN compared with that in healthy volunteers. Cell culture assay results suggested that hsa_circ_0003928 expression is upregulated upon HG stimulation in a concentration- and time-dependent manner *in vitro* [91]. Silencing hsa_circ_0003928 could restore impaired cell viability and attenuate cell apoptosis, ROS levels, and inflammatory cytokine production in HK-2 cells under HG conditions. In addition, miR-151-3p expression was significantly downregulated in HG-cultured HK-2 cells; such expression was restored by knocking down hsa_circ_0003928 *in vitro*. MiR-151-3p expression was also downregulated in the serum of patients with DN. A luciferase reporter assay suggested that there are direct interactions between hsa_circ_0003928 and miR-151-3p as well as between miR-151-3p and Anxa2. Considering the regulatory role of the hsa_circ_0003928/miR-151-3p axis on Anxa2 expression and the rescue functions of miR-151-3p inhibition over hsa_circ_0003928 knockdown, hsa_circ_0003928 might function in DN development by promoting inflammation and cell apoptosis, partly through the miR-151-3p/Anxa2 axis [91]. The upregulation of hsa_circ_0003928 expression in the serum of patients with DN implies that this circRNA might serve as a non-invasive diagnostic biomarker for DN, thereby warranting further investigation.

#### CircACTR2

Recent studies have illustrated that the microinflammatory state is crucial for the development of diabetic kidney disease [92, 93]. Pyroptosis is distinct from apoptosis, and is a form of gasdermin D (GSDMD)-dependent inflammatory programmed cell death that is involved in tubular injury in diabetes [94]. To investigate the potential role of circRNAs in HG-induced inflammation and pyroptosis in renal tubular epithelial cells, HK-2 cells were cultured with different concentrations of glucose. In addition, VX-765, a small molecule caspase-1 inhibitor that prevents inflammasome activation and pyroptosis, was used to explore the circRNAs involved in inflammation and pyroptosis under glucose exposure *in vitro* [45]. Microarray analysis of circRNA expression proﬁles identified 117 differentially expressed circRNAs between the NG and HG groups and 157 between the HG and HG + VX-765 groups (fold change ≥ 2.0, p < 0.05). Among the dysregulated circRNAs, circACTR2 (hsa_circRNA_102747, hsa_circ_0008529) expression was markedly upregulated in HG-treated HK-2 cells, which could be reversed by treatment with VX-765 [45]. An *in vitro* experiment using flow cytometry analysis as well as PI uptake and LDH release measurements revealed that knocking down circACTR2 significantly suppressed pyroptosis. Simultaneously, suppressing circACTR2 expression could reduce the generation of Col-IV and FN and inhibit the release of mature IL-1β into the medium in HG-stressed HK-2 cells *in vitro*. Therefore, dysregulation of circACTR2 could influence pyroptosis, inflammation, and ﬁbrosis induced by HG in proximal tubular cells [45]. However, the mechanism by which circACTR2 exerts these functions *in vivo* remains unclear.

#### CircEIF4G2

In a study to elucidate the role of circEIF4G2 in DN, a *db/db* mouse model of T2D and NRK-52E cells were used to determine the effects of HG stimulation. Xu et al. found that the expression of circEIF4G2 in the kidneys of *db*/*db* mice was significantly higher than that in *db*/*m* mice [95]. In addition, the levels of circEIF4G2 were significantly higher in HG-stimulated NRK-52E cells than in the control group. However, the expression levels of miR-218 displayed opposite trends in mouse kidneys and cell lines. The downregulation of SERBP1, TGF-β1, Col-I, and FN expression induced by circEIF4G2 silencing could be reversed by co-transfection with miR-218 inhibitors *in vitro*. Knockdown of SERBP1 further suppressed the expression of these fibrosis-related proteins. The predicted direct interactions between miR-218 and circEIF4G2 or SERBP1 were validated using luciferase assays. Thus, circEIF4G2 might regulate the generation of fibrosis-related proteins *in vitro* via the miR-218/SERBP1 pathway [95].

### CircRNAs in podocytes

#### Circ_0000285

Several studies have been conducted to investigate the functions of dysregulated circRNAs in mesangial cells during the progression of DN; however, studies focusing on podocytes are rare. Yao et al. found that the expression of circ_0000285 was remarkably elevated in mouse DN kidney tissues *in vivo* and that its expression in mouse podocytes was also increased under HG exposure *in vitro* [96]. After overexpressing circ_0000285 *in vitro*, the proliferative ability of podocytes was repressed, whereas apoptosis was enhanced. Simultaneously, circ_0000285 blocked the cell cycle at the G1 phase in podocytes. Pull-down assay results indicated that miR-654-3p could be enriched by a circ_0000285-specific probe and that miR-654-3p mimics suppressed the luciferase activity of WT circ_0000285, suggesting a direct interaction between miR-654-3p and circ_0000285. After knocking down circ_0000285, the proliferative ability of podocytes was increased, and apoptosis was suppressed; these effects could be reversed by miR-654-3p downregulation. The levels of MAPK6, TNF-α, IL-6, and IL-1β were reduced following silencing of circ_0000285 and increased by inhibiting miR-654-3p [96]. Considering the direct interaction between miR-654-3p and MAPK6, circ_0000285 might function through the miR-654-3p/MAPK6 axis to regulate the release of inflammatory cytokines in DN podocytes *in vitro* [96]. However, the pathophysiological mechanisms by which circ_0000285 regulates inflammatory molecule biosynthesis *in vivo* require further investigation.

### CircRNAs in exosomes

#### Exosomal circRNAs

To elucidate the molecular mechanisms underlying DN, Ling et al. studied exosomes isolated from glomerular endothelial cells (GECs) under HG and NG conditions. High throughput sequencing was used to screen differentially expressed exosome-trafficked circRNAs between the two groups [97]. GECs under HG conditions produced more exosomes than GECs under NG conditions, with 217 upregulated and 484 downregulated circRNAs between the two groups. KEGG analysis indicated that the dysregulated circRNAs were most enriched in the PI3K/AKT and RAS signaling pathways. QRT-PCR was used to validate the downregulation of mmu_circ_0001605 (circRNF169) and mmu_circ_ 0000372 (circSTRN3) expression in exosomes produced by GECs under HG conditions compared with those produced by GECs under NG conditions. The expression of circRNF169 and circSTRN3 was downregulated in HG-treated GECs compared with that in NG-treated GECs. When cocultured with mesangial cells, exosomes generated from GECs under different conditions could be absorbed by mesangial cells. Interestingly, exosomes produced by HG-treated GECs were endocytosed more frequently, resulting in the inhibition of circRNF169 and circSTRN3 expression and of the proliferative ability of mesangial cells.

Ling et al. performed additional experiments to elucidate the molecular mechanisms underlying these differences and found that exosomes produced by HG-treated GECs promoted the expression of α-SMA in cocultured mesangial cells and of FN and Col-IV in the culture medium supernatant. *In vitro* knockdown of circRNF169 and circSTRN3 in mesangial cells, using double-stranded siRNAs, inhibited cell proliferation and increased the expression of α-SMA and Col-IV in the supernatant, thus inducing EMT. Furthermore, *in vitro* overexpression of circRNF169 and circSTRN3 in mesangial cells exhibited opposing effects and promoted the migration of mesangial cells [97]. Thus, targeting exosomes produced by GECs and exosome-trafficked circRNAs, such as circRNF169 and circSTRN3, could be a potential therapeutic strategy to intervene in the exosome-mediated crosstalk between mesangial cells and GECs to inhibit renal fibrosis [97, 98].

## Perspectives

As one of the most common causes of end-stage renal diseases (ESRD) and chronic renal failure in patients with diabetes, DN is pathologically characterized by albuminuria, mesangial cell proliferation, ECM accumulation, glomerular hypertrophy, and kidney fibrosis or failure [72, 99]. The molecular mechanisms underlying the occurrence and progression of DN are complex and include glucose metabolism disorders, inflammasome activation, oxidative stress, cell apoptosis, cellular senescence, and renal hemodynamic changes [14, 100, 101]. CircWBSCR17, circ_0000712, circITCH, circLRP6, circACTR2, and circ_0000285 are involved in inflammasome activation in DN [45, 82, 86, 89, 90, 96]. Meanwhile, circAKT3, circWBSCR17, hsa_circ_0003928, and circ_0000285 can regulate apoptosis in this disease [73, 90, 91, 96]. Almost all of the circRNAs included in the present review participate in ECM accumulation and fibrosis of the kidneys by modulating the expression levels of related proteins. The functions and expression patterns of the circRNAs included in the present review are summarized in Table 1

Despite the extensive research efforts summarized in the present review, there are still some evident gaps in our knowledge. There remains a lack of information on the role of dysregulated circRNAs in the progression of other molecular pathologies, such as increased oxidative stress and cellular senescence. The roles of circRNAs in GECs and other glomerulus cell types are also unclear. Except for their function as endogenous miRNA sponges, any other participation of circRNAs in the development and progression of DN remains unknown. Hypothetically, circRNAs may interact with RBPs or serve as translational templates in DN. These gaps warrant further research on circRNAs.

**Table 1:** Dysregulated circRNAs and their functional mechanisms in diabetic nephropathy.

CircRNA	Gene symbol	Cell type	Expression changes	Functions	Possible mechanism	Ref.
**CircHIPK3 (hsa_circ_0000284)**	HIPK3	Mesangial cell	Up	Promote proliferation of mesangial cells and expression of TGF-β1, Col-I, and FN	Regulating miR-185	70
**CircRNA_15698 (mmu_circ_0015698)**	-	Mesangial cell	Up	Promote expression of TGF-β1, FN, Col-I, and Col-IV	Regulating miR-185/TGF-β1 axis	72
**CircAKT3**	AKT3	Mesangial cell	Down	Inhibit the apoptosis of mesangial cell and expression of FN, Col-I, and Col-IV	Regulating miR-296-3p/E-cadherin axis	73
**Circ_0000064 (hsa_circ_0000064)**	-	Mesangial cell	Up	Promote proliferation of mesangial cell and expression of FN, Col-I, and Col-IV while inhibiting cell apoptosis	Regulating miR-143	76
**Circ_0123996**	-	Mesangial cell	Up	Promote proliferation of mesangial cells and expression of FN and Col-IV	Regulating miR-149-5p/Bach1 axis	77
**CircLRP6**	LRP6	Mesangial cell	Up	Promote proliferation, oxidative stress, the secretion of FN, Col-IV, IL-6, IL-1β, and TNF-α in mesangial cells via TLR/NF-κB pathway	Regulating miR-205/HMGB1 axis	82
**CircLARP4**	LARP4	Mesangial cell	Down	Inhibit proliferation of mesangial cells, enhance cell apoptosis and decrease the expression levels of FN, Col-I and Col-IV	Regulating miR-424	83
**Circ_0080425**	-	Mesangial cell	Up	Promote proliferation, cell cycle progression and expression of FN, Col-IV, and TGF-β in mesangial cell	Regulating miR-24-3p/FGF11 axis	84
**Circ_0000491 (mmu_circrna_0000491)**	Homer1	Mesangial cell	Up	Promote expression of vimentin, FN, α-SMA, Col-I, III and IV while suppressing expression of E-cadherin	Regulating miR-101b/TGFβRI axis	85
**Circ_0000712 (mmu_circrna_0000712)**	-	Mesangial cell	Up	Promote apoptosis, upregulate expression levels of IL-1β, IL-6, TNF-α and fibrosis-related protein FN, Col-I, Col-IV, increase ROS generation and LDH activity as well as decrease SOD activity in mesangial cells	Regulating the miR-879-5p/SOX6 axis	86
**Circ_0037128**	-	Mesangial cell	Up	Promote proliferation, cell cycle progression and expression of FN and TGF-β in mesangial cell	Regulating miR-17-3p/AKT3 axis	87
**CircITCH**	ITCH	Mesangial cell	Down	Reduce viability and inhibit migration of mesangial cells, suppress the expression levels of Col-I, α-SMA, FN, IL-6, IL-1β, MPO and TNF-α	Regulating miR-33a-5p/SIRT6 axis	89
**CircRNA_010383**	Akap7	Mesangial cell and tubular cells	Down	Inhibit the synthesis of FN, Col-I, α-SMA and PCNA in mesangial cell; inhibited the synthesis of FN, Col-I, α-SMA and promote the synthesis of E-cadherin in tubular cells	Regulating miR-135a/TRPC1 axis	15
**CircWBSCR17**	-	Tubular cells	Up	Inhibit cell proliferation of kidney tubular cells and promote apoptosis as well as expression of TNF-α, IL-6, IL-1β, FN, Col-IV and Col-I	Regulating miR-185-5p/SOX6 axis	90
**Hsa_circ_0003928**	-	Tubular cells	Up	Inhibit cell viability and promote inflammation as well as cell apoptosis of kidney tubular cells	Regulating miR-151-3p/Anxa2 axis	91
**CircACTR2 (hsa_circRNA_102747, hsa_circ_0008529)**	ACTR2	Tubular cells	Up	Promote pyroptosis and expression of Col-IV, FN and IL-1β in tubular epithelial cells	-	45
**CircEIF4G2**	-	Tubular cells	Up	Promote expression of TGF-β1, Col-I and FN in tubular cells	Regulating miR-218/SERBP1 axis	95
**Circ_0000285**	-	Podocytes	Up	Inhibit proliferation, promote cell apoptosis and expression of TNF-α, IL-6 and IL-1β, block cell cycle at G1 phase in podocytes	Regulating miR-654-3p/MAPK6 axis	96
**CircRNF169 and circSTRN3 (mmu_circ_0001605 and mmu_circ_0000372)**	-	Glomerular endothelial cells and mesangial cells	Down	Exosomes promote the expression of α-SMA in cocultured mesangial cells and the expression of FN and Col-IV in the culture medium supernatant	Produced by glomerular endothelial cells, absorbed by mesangial cells	97

Although many studies to date have focused on the role of circRNAs *in vitro*, few *in vivo* investigations have been conducted. The functional roles of circRNAs in DN therefore need to be further validated in animal models and in clinical studies. While the expression level of circHIPK3 was upregulated in diabetic retinas and retinal endothelial cells under HG conditions [69], it was downregulated in primary HAECs from patients with diabetes and in HUVECs cultured in the presence of HG [71]. Meanwhile, the expression of circHIPK3 increased in mesangial cell lines under HG conditions [70]. Therefore, the expression levels of circHIPK3 are dynamically regulated depending on the cellular context, even in the same disease condition. A similar inconsistency has also been observed in studies of various cancers. Although circHIPK3 serves as an oncogene in hepatocellular carcinoma and colorectal cancer, it acts as a tumor suppressor in osteosarcoma and bladder cancer [102, 103]. Another example is circMOT1 expression, which is upregulated in cervical cancer and gallbladder cancer but downregulated in bladder cancer, renal cell carcinoma, and ovarian cancer [53]. These discrepancies might result from the different tissues and cell lines used in different studies as well as from tissue- and cell-specific features of circRNAs. Based on these inconsistencies, the choice of cell or tissue system used to study these circRNAs is of the utmost importance.

Exosomes, which are formed by an intracellular endocytic trafficking pathway, are important modulators of cell-to-cell and organ communication [104, 105]. By transmitting their cargos, such as proteins, mRNA, and non-coding RNAs, exosomes can participate in the development of diabetes and concomitant complications [106]. Wu et al. found that exosomes from HG-treated GECs could transfer TGF-β1 mRNA to activate mesangial cells, thereby promoting renal fibrosis [98]. HG-cultured HUVECs can modulate the senescence of vascular smooth muscle cells by transmitting circRNA-0077930 via exosomes [107]. In their recent study focusing on diabetes-induced retinal vascular dysfunction, Liu et. al found that the expression levels of circPWWP2A, acting as an endogenous miR-579 sponge, were increased in pericytes but not in endothelial cells under diabetes-related stresses *in vitro*. Further studies on the pericyte-endothelial cell crosstalk have suggested that exosomes generated by pericytes could transfer circPWWP2A to endothelial cells to indirectly modulate their biology [108]. Out of the literature summarized in the present review, only one study has specifically addressed the potential roles of exosome-trafficked circRNAs in cell-to-cell communication in DN [97]. More research on the roles of circRNAs that are trafficked by exosomes in crosstalk between various cell types in the DN microenvironment is urgently needed and could be of great clinical relevance.

Considering their abundance, stability, and specificity, circRNAs in the circulating blood are ideal non-invasive biomarkers for various diseases, including diabetes [5, 41, 109, 110]. Yang et al. suggested that plasma hsa_circRNA_102893 might serve as a potential novel and stable non-invasive biomarker for detecting early gestational diabetes mellitus [111]. In addition, upregulated hsa_circ_0054633 in the peripheral blood could be a potential biomarker for pre-diabetes and T2D [112] as well as for gestational diabetes mellitus [113]. Fang et. described the value of circANKRD36 in the peripheral blood leucocytes for screening chronic inflammation in patients with T2D [114]. However, there is insufficient research on circRNA biomarkers in the serum for DN. Although the upregulation of hsa_circ_0003928 and the corresponding downregulation of miR-151-3p in the serum of patients with DN have been validated, their potential as diagnostic biomarkers has not been sufficiently explored [91]. Thus, further studies should be conducted to address the potential application of serum circRNAs as biomarkers for the diagnosis and prognosis of DN.

Urine is another easily obtained biofluid for performing non-invasive analyses of biomarkers for kidney diseases, including DN [115]. The content of urine exosomes is a better candidate than whole urine for DN biomarker analyses because urine exosomes are primarily released by cells in the nephrons and collecting ducts, and blood exosomes cannot pass through the glomerular membrane into the urine [116, 117]. Recently, Zhao et. al suggested that urinary exosomal miRNA-4534 was a promising diagnostic biomarker for DN progression [118]. Xie et al. found that exosomal miR-362-3p, miR-877-3p, miR-150-5p, and miR-15a-5p could serve as potential novel biomarkers for early DN [119]. Likewise, circRNAs could be enriched in the exosomes and are therefore ideal candidate biomarkers for kidney diseases [41]. Ma et al. found 54 circRNAs whose expression was upregulated and 6 circRNAs whose expression was downregulated in urinary exosomes in patients with idiopathic membranous nephropathy compared with those of healthy controls [120]. Similarly, 450 upregulated and 26 downregulated circRNAs were identified in urinary exosomes of patients with immunoglobulin A nephropathy compared to those of healthy controls [121]. These studies validated the feasibility and eligibility of urinary exosomal circRNAs as biomarkers for kidney diseases. However, there have been no related studies on the diagnostic potential of urinary exosomal circRNAs in DN, and thus, this should be pursued in the future. It is speculated that dysregulated circRNA expression in urinary exosomes of patients with DN could serve as a valuable diagnostic biomarker for early DN, contributing to the early detection of DN and providing an opportunity to prevent progression to ESRD.

In conclusion, circRNAs are essential modulators of the initiation and development of DN. The functions of circRNAs in DN are gaining considerable interest and have become the focus of diabetes research. However, many important yet challenging questions remain to be answered regarding these RNA rings and their roles in DN. More studies are warranted to further explore the roles of circRNAs in the pathophysiological processes of DN; they might serve as a foundation for developing novel diagnostic and therapeutic approaches.
